# Assessing the impact of thermoregulatory mineral supplementation on thermal comfort in lactating Holstein cows

**DOI:** 10.1016/j.vas.2024.100363

**Published:** 2024-05-23

**Authors:** Rafael Felini, Damiano Cavallini, Giovanni Buonaiuto, Tiago Bordin

**Affiliations:** a Curso de Agronomia, Faculdade CESURG Marau, Avenida Júlio Borella, 1968, Centro, CEP 99150-000, Marau, RS, Brasil; b Department of Veterinary Sciences, University of Bologna, Ozzano dell'Emilia, 40064, Bologna, Italy

**Keywords:** Heat stress, Animal welfare, Physiological response

## Abstract

Heat stress (HS) occurs when animals are enable to effectively dissipate excessive body heat, potentially affecting their welfare and productivity. Several tools can be used to mitigate HS in dairy cows. The aim of this study was to evaluate the effect of thermoregulatory mineral supplementation on dairy cows’ physiological response to HS and reproductive status. Thirty pluriparous Holstein dairy cows (2.8 ± 0.3 lactation), from a semi confinement production system (freestall barn), were enrolled in a 35 days duration experiment, and divided into two groups: 15 cows receiving a thermoregulatory mineral mixture containing calcium, sodium, chlorine, and potassium (200 g/day; TRT); and, 15 cows that did not receive any type of supplement (CON). Data on respiration rates (RR), rectal temperature (RT), milk yield, barn temperature, relative humidity, and temperature and humidity index (THI) were obtained on weekly evaluations (D7, D14, D21, D28, and D35). ANOVA and correlation analysis were used to compare variables between groups, and physiological and climate variables, regardless of group. Related to farm's protocols, on D0 and D35, all cows were submitted to US evaluation and categorized as inseminated, pregnant, and not pregnant, and this data analysed using Fisher's exact test. Cows in the TRT group had lower RT, compared to the CON group (38,8 °C and 39,0 °C, respectively; *P* = 0.0147), however both averages were within physiological range. As to physiological variables, stronger positive correlations were found between RR and RT with barn temperature and THI. In this study, the thermoregulatory mineral supplement did not significantly affect physiological responses to HS or reproductive status.

## Highlights

• Thermoregulatory mineral mixture did not show effect under moderate HS conditions
• Other tools to mitigate HS may play a more important role than mineral supplement
• Even mild and moderate HS can elevate respiratory rate and rectal temperature

## Introduction

Ruminant production is a vital and complex system that supplies food for humans, but it currently faces several challenges. These challenges include antibiotic resistance, environmental degradation, feed scarcity, pandemic diseases, and the imminent threat of climate change (Place et al., 2022; Masebo et al., 2023). Climate change has the potential to affect livestock systems in various ways. One significant effect is the reduction in pasture growth and yield, accompanied by alterations in its composition, which may occur gradually due to the rising concentration of CO_2_ (Yamori et al., 2014). An immediate concern is the increase in average temperature, which can negatively affect the thermal comfort of dairy cattle. The thermal comfort zone of Holstein dairy cows typically ranges from 0 to 20°C (Desrousseaux, 2021), thereby increasing the risk for heat stress (HS) for animals in tropical and subtropical regions worldwide. In southern Brazil, HS alone accounts to a substantial 21 % loss in milk production (Peretti et al., 2022).

Climate and environment changes are also a pressing concern for consumers and organizations focused on animal welfare, pressuring for more sustainable livestock practices. Implementing effective thermoregulatory strategies within dairy production systems is crucial not only from an economic perspective but also from social and environmental standpoints (Leiva et al., 2017).

As global temperatures rise and HS increasingly affects livestock, the sector faces growing concerns regarding both animal welfare and productivity (quantity and quality; Buonaiuto et al., 2024). HS occurs when animals can no longer efficiently dissipate the excessive body heat they generate. High temperatures and humidity are the primary contributors to HS, resulting in animals losing their capacity for thermoregulation through evapotranspiration (Desrousseaux, 2021). Additionally, prolonged exposure to excessive heat negatively impacts reproduction and overall homeostasis. This leads to reduced feed intake and the development of metabolic disorders like acidosis and lameness (de Andrade Ferrazza et al., 2017). Heat-stressed dairy cows often exhibit compromised health status, fertility disorders, and increased mortality rates (Min et al., 2019).

Several strategies have emerged as potential solutions, which can be categorized into three main approaches: (i) physical modification of the environment; (ii) breeding of thermos-tolerant animals; and, (iii) improvements in feeding and nutritional management (Fournel et al., 2017; Conte et al., 2018). Within strategic nutritional management, the use of supplements such as vitamins, concentrates, fat, botanicals, and additives, has shown promising results, enhancing ruminants’ body temperature efficient regulation (Colombo et al., 2019; Oh et al., 2021; Vittorazzi et al., 2022; Giorgino et al., 2023). Another approach to mitigate HS through nutritional intervention is dietary mineral supplementation. Minerals play a crucial role in the overall wellbeing of dairy cows, particularly during reproductive and lactation periods. When HS is present, minerals demands increase, including sodium (Na) and potassium (K), to facilitate proper thermoregulation (Guesine et al., 2023).

Therefore, our study aimed to evaluate the effects of a commercially available thermoregulatory mineral supplementation on the physiological responses, milk yield, and reproductive status of Holstein dairy cows.

## Materials and methods

The trial was conducted on a commercial dairy farm located in the Santa Cecília municipality, Rio Grande do Sul state, Brazil (28° 7′ 51″ S, 51° 55′ 36″ W). All experimental procedures were approved by the Ethics Committee of CESURG Marau Faculty (protocol number 02.2023).

### Cows, housing, and diets

Thirty (30) lactating pluriparous Holstein cows, with an average milk production of 29.5 L/day, were selected based on weight (640.5 ± 5.2 kg), days in milking (90 ± 10), and age (58.2 ± 0.1 months). Prior to the experimental period, pregnancy diagnosis was conducted using a portable ultrasound device (Infinit IV, Ultramedic®, Porto Alegre, RS, Brazil), and timed artificial insemination (TAI) were performed. Subsequently, the cows were randomly assigned to two groups: one comprising seven cows inseminated on D0, five not pregnant, and three 90 days pregnant, totaling 15 cows per group. The control group (CON) received the regular diet, while the treatment group (TRT) received a mineral supplement (TermoMix®; Alger Nutrition LTDA®) containing calcium (90 to 110 g/kg), chlorine (250 g/kg), sodium (165 g/kg), and potassium (125 g/kg), administered at a dose of 200 g per cow per day, as per the manufacturer's instructions.

During the day, cows were housed in a 900 m^2^ freestall barn. Each cow had access to an individual stall measuring 2.8 m^2^, which was cleaned twice daily and bedded with wood shavings for comfort. Potable water was provided *ad libitum* via four troughs (1.5 m2 each), and their diet was dispensed along a 60 m feeding line, with partitions every 85 cm to ensure cows were separated while feeding. To mitigate heat stress (HS), the farm installed a forced-ventilation system in the barn comprising six fans, which generated a wind velocity of 3 m/s (Weg® 0.5 cv 6P 48 1F 110-127/220-254 V 60 Hz IC418 – TEAO). Additionally, thirty sprinklers were evenly distributed along the feeding line, positioned at a height of 2.5 m from the ground, with water flow rates ranging from 2 to 4 L/min. These fans and sprinklers operated simultaneously during feeding times.

Animals were fed a total mixed ration (TMR) twice daily (at 8 a.m. and 2 p.m.) using a mixer wagon (Haramaq® HQ 300). The diet composition (Table 1) was formulated according to the guidelines provided by NRC (2007). For TRT cows, TMR was top-dressed with 100 g of the mineral supplement at each feeding time. Cows were restrained in headlock within individual stalls for one hour during both feeding times to ensure complete ingestion of the supplement. Workers used shovels to push the TMR closer to the feeding line four times daily (twice in the morning and twice in the afternoon). Leftover feed was weighed daily to estimate the feed intake.

**Table 1:** Ingredient composition of the experimental diets supplied to Holstein dairy cows supplemented or not with a thermoregulatory mineral mixture.

Ingredient	CON		TRT	
	Kg of FM	Kg of DM	Kg of FM	Kg of DM
Tifton-85 pasture	19	3.420	19	3.420
Maize silage	30	9.879	30	9.879
Ryegrass hay^a^	0.8	0.720	0.8	0.720
Ground corn	3.43	2.981	3.43	2.981
Integral soybean meal	2.200	1.964	2.200	1.964
Extruded soybean meal	0.640	0.598	0.640	0.598
Soybean hull	0.640	0.579	0.640	0.579
Wheat meal	0.640	0.577	0.640	0.577
Urea	0.100	0.099	0.100	0.099
Sodium bicarbonate	0.128	0.128	0.128	0.128
Magnesium oxide	0.040	0.040	0.040	0.040
Mineral nucleus^b^	0.400	0.396	0.400	0.396
Mycotoxin adsorbent^c^	0.008	0.007	0.008	0.007
Yeast	0.010	0.009	0.010	0.009
Mineral treatment (TermoMix®)^d^	-	-	0.200	0.196
Total	58.036	21.397	58.236	21.593

CON: control group (without TermoMix®); TRT: treatment group (supplemented with Termo-Mix®); FM: fresh matter; DM: dry matter.

a Evaluated for its quality and the absence of undesirable weeds [18]

b 155–200 g/kg of calcium, 51 mg/kg of cobalt, 700 mg/kg of copper, 6 mg/kg of chrome, 21 g/kg of sulfur, 50 g/kg of phosphor, 700 mg/kg of fluorine, 40 mg/kg of iodine, 35 g/kg of magnesium, 1.289 mg/kg of manganese, 24 g/kg of potassium, 18 mg/kg of selenium, 80 g/kg of sodium, 2.700 mg/kg of zinc, 40 mg/kg of biotin, 300.000 IU/kg of vitamin A, 60.000 IU/kg of vitamin D3, 2.200 IU/kg mg of vitamin E, 4.000 mg/kg of niacin, 600 mg/kg of sodium monensin

c Added to avoid mycotoxin adverse effect on the experimental animals [19]

d 90-110 g/kg of calcium, 250 g/kg of chlorine, 165 g/kg of sodium, 125 g/kg of potassium; TMR chemical composition of TRT: DM: 37.1 %, Crude protein: 16.5 %, Neutral detergent fiber: 36.9 %, Acid detergent fiber: 21.4 %, Starch: 22 %, Ash: 8.9 %, Fatty Acids: 2.68 %, Calcium: 0.75 %, Phosphor: 0.44 %, Magnesium: 0.37 %, Sodium: 0.54 %, Chlorine: 0.59 %, Sulfur: 0.24 %, Dietary cat-ion-anion balance: 373 mEq/kg.

Following the second milking session, cows were led to a Tifton-85 grass pasture area located 220 m from the freestall barn, covering an area of 5.5 hectares. At 6 a.m., all cows were moved from the pasture to the waiting area of the milking room. The paddock was equipped with four potable water troughs, while the waiting room had one trough, each with a capacity of 1.5 m^2^.

### Sample collection and analyses

Once a week, a sample of TMR (500 g) was collected immediately after mixing and frozen to preserve its chemical characteristics for subsequent analysis. The TMR samples were sent to a laboratory for dry matter (DM) and chemical analysis, with detailed methodologies published in previous research (Cavallini et al., 2023).

During the experiment period, four hygrometers were placed inside the barn to collect daily data on air temperature and relative humidity using a AK28® digital hygrometers (Akso, São Leopoldo, RS, Brazil). These data were utilized to calculate the temperature and humidity index (THI), according to the equation of NRC (1971):

THI=(1.8×Ta+32)−(0.55−0.0055×RH)×(1.8×Ta−26)

where Ta represents the air temperature and RH represents the relative humidity. The THI values were then categorized as follows: thermoneutrality (TN, 60 ≤ THI < 67), mild heat stress (Mild HS, 67 ≤ THI < 72), moderate heat stress (Moderate HS, 72 ≤ THI < 80), and severe heat stress (Severe HS, 80 ≤ THI < 86) conditions (Collier et al., 2012).

Cows were milked twice a day, at 6:40 a.m. and 6:30 p.m., using a pipeline mechanical system (Milkparts®, Westfália, RS, Brazil) in a double-6 tandem milking room. Milk yield data were recorded individually once a week over the course of five weeks using the infrared meter (MP 700 Metter, Milkparts®, Westfália, RS, Brazil) of the milking system. Production was determined by summing the yields from both milkings and calculating the average milk yield for the groups on each sampling day.

In addition to environment climate data, the physiological response of cows to HS was evaluated. Respiratory rate (RR) was assessed once a week by counting breathing movements in one minute (mov/min), consistently at 10 a.m. over the same five-week period. Following RR assessment, rectal temperature (RT) was measured using digital thermometer (Domotherm, digital clinical thermometer, Incoterm®).

During daytime hours, cows were observed in the barn for signs of estrus, including mounting behavior and vaginal discharge. A trained individual inspected the cows daily, and those exhibiting signs of estrus were inseminated. At the end of the study (D35), reproductive status was evaluated by a veterinary using ultrasonography (Infinit IV, Ultramedic®, Porto Alegre, RS, Brazil) and cows were classified as inseminated, pregnant, or not pregnant.

### Statistical analysis

Data normality and homoscedasticity were verified through Shapiro-Wilk's and Levene's test, respectively, and were analysed comparing CON and TRT groups. Each cow was considered as an experimental unit, within groups.

Initially, a repeated measures ANOVA was performed considering group (CON and TRT) and sampling day (D7, D14, D21, D28, and D35) as fixed effects, and animal as random effect. Tukey's test was used for pairwise comparison between groups, days, and their interaction. As for reproductive status (inseminated, pregnant, and not pregnant), groups were compared using Fisher's exact test.

Physiological response data (RR, RT, and milk yield) and barn's climate data (temperature, relative humidity, and THI) were submitted to correlation analysis, regardless of experimental group. Magnitude of correlation coefficient was interpret as proposed by Schober et al. (2018): negligible (0.00 – 0.10); weak (0.10 – 0.39); moderate (0.40 – 0.69); strong (0.70 – 0.89); and, very strong (0.90 – 1.00).

Analyses were executed in JMP Pro 14 software (SAS Institute Inc.), and statistical significance was achieved when P < 0.05.

## Results

### Physiological responses and milk yield

**Fig. 1:**
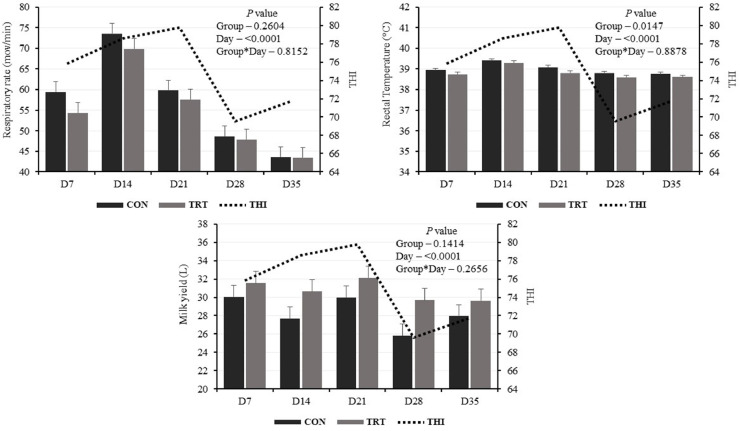
Physiological responses and milk yield of Holstein dairy cows fed with diets containing or not a thermoregulatory mineral supplement (TermoMix®), on days 7, 14, 21, 28, and 35 (D7, D14, D21, D28, and D35, respectively) of the experimental period.

CON: Control group; TRT: Treatment group; THI: Temperature and humidity index

Through experimental period THI ranged from 67 (thermoneutrality), on the morning of D32, to 83 (severe HS), on the afternoon of D14. At the time of evaluation (10 a.m.), THI was mild on D28, moderate on D7, D14, and D35, and severe on D21 (Fig. 1).

There was a significant difference between groups regarding RT (CON: 39.0°; TRT: 38.8°C; *P* = 0.0147), while RR and milk yield only showed difference between sampling days (*P* < 0.0001; Fig. 1). No differences were observed within sampling days between groups or interaction of these effects.

Milk yield was only weak and positive correlated with barn temperature and THI (Table 2). Moderate and positive correlation was found between RR × RT, RR × barn temperature, RR × THI, RT × barn temperature, and RT × THI, and very strong positive correlation between barn temperature × THI. Relative humidity presented negative moderate correlation with RR, RT, and THI, and strong with barn temperature.

**Table 2:** Correlation matrix between physiological responses and milk yield of Holstein dairy cows, and freestall barn's temperature, relative humidity, and temperature and humidity index (THI).

Variables	Respiratory rate^1^	Rectal temperature^a^	Milk Yield^a^	Temperature^a^	Relative humidity^b^	THI^a^
Respiratory rate	1.0000	0.3979⁎⁎	0.0355	0.6318⁎⁎	-0.4382⁎⁎	0.5794⁎⁎
Rectal temperature		1.0000	-0.1194	0.4809⁎⁎	-0.4062⁎⁎	0.4251⁎⁎
Milk Yield			1.0000	0.1692*	-0.0806	0.1937*
Temperature				1.0000	-0.7000⁎⁎	0.9838⁎⁎
Relative humidity					1.0000	-0.6020⁎⁎
THI						1.0000

THI: temperature and humidity index

a Pearson's r

b Spearman's ρ

⁎ *P* < 0.05

⁎⁎ *P* < 0.001

### Reproductive status

**Fig. 2:**
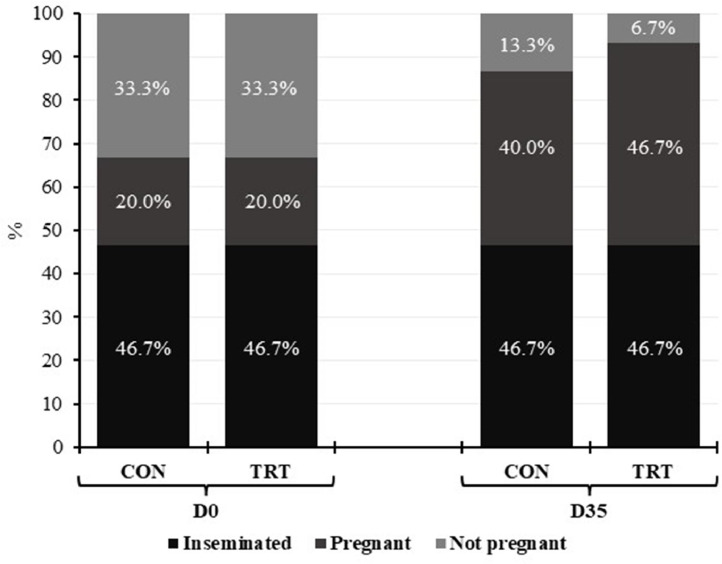
Reproductive status of Holstein cows fed diets containing or not thermoregulatory mineral mixture (TermoMix®), at the beginning (D0) and at the end (D35) of the experimental period.

CON: Control group; TRT: Treatment group

No difference was observed between groups regarding reproductive status at the end of the study (Fig. 2).

## Discussion

This comprehensive study aimed to evaluate the effects of a commercially available thermoregulatory mineral supplementation, on physiological responses, milk production, and reproductive status of Holstein dairy cows.

### Physiological responses and milk yield

The use of the thermoregulatory mineral supplement reduced RT in TRT cows, however, averages from both groups remained within the physiological range (38.5 – 39.5; Feitosa, 2020), and other variables were not influenced by it. Minerals play important roles in the maintening of physiological status of ruminants, especially in highly productive dairy cows (Sammad et al., 2020). HS may induce negative energy balance (NEB) in lactating cows due to reduced feed intake and increased energy expenditure in maintaing thermoregulation (Conte et al., 2018). Thermic stress leads to mineral losses because of the elevated body fluid loss caused by excessive excretion when animals cannot dissipate heat properly (Leiva et al., 2017).

The rise in RR and RT is a known mechanism in HS physiological response (Becker et al., 2020); nevertheless, maximum averages of these variables were observed at D14, while the maximum THI was noted at D21. It is possible that another factor interfered with the elevation seen in RR and RT at D14, such as a change in overall handling or other stressful factors.

Trace minerals such as chromium and selenium have been recognized as potential reducers of HS (Leiva et al., 2017, Weng et al., 2018). However, macromineral nutrients such as Na and K can also alleviate thermic stress in ruminants, as they demand more Na+ and K+ cations due to elevated urinary excretion. The thermoregulatory supplement was composed of Na, K, Cl, and Ca, which likely contributed to increased feed intake and milk yield performance in cows (NRC, 2021). Additionally, increased dietary calcium has been shown benefits lactating cows by preventing excessive and eventual bone calcium mobilization (Gaignon et al., 2019; Caixeta and Omontese, 2021).

Throughout this study, cows were kept indoors for most of daytime, shielded from direct sunlight, and severe HS occurred only on one sampling day. It is well-established that the duration and intensity of HS exposure are crucial determinants in altering physiological response (as reviewed by Hoffmann et al., 2020). Therefore, it is necessary to evaluate the efficacy of this mineral supplement on cows under prolonged and more intense heat loads than those encountered in this study.

The forced acclimation of animals unadapted to warm environments can reduce dry matter intake, rumen motility, saliva production, and salivary bicarbonate production (i.e., natural ruminal buffering; Burhans et al., 2022). Additionally, heat-stressed dairy cows often exhibit metabolic and endocrine alterations. Elevated blood urea nitrogen (BUN) levels may result from failures in ammonia transformation into microbial protein (Hou et al., 2021). Beta-OH butyrate, an important ketone body, is also higher in thermally stressed animals due to the mobilization of non-esterified fatty acids (NEFA) in the liver to compensate for reduced nutrient intake (Turk et al., 2015; Al-Qaisi et al., 2019). Insulin blood levels often increase in heat-stressed animals as the pancreas enhances its secretion under HS conditions, leading to tissue insulin resistance. These changes may result in milk yield depression, however, this study, did not perform metabolic analyses, and milk yield showed only a weak positive correlation with temperature and THI. Evaluating a larger number of cows over an extended period may be more effective in detecting the influence of HS on production. A significantly negative correlation (r = – 0.187) was found between these two parameters in Holstein cows in Ukraine, using environmental data collected every hour, over 123 days, with THI range between 47.1 and 84.7 (Mylostyvyi & Chernenko, 2019). Data from over 5,000 Holstein cows from three dairy farms, over four years, showed a reduction of 14.3 % in daily milk yield from low (<70) to high (>80) THI (Nasr & El-Tarabany, 2017). However, in a 10-year retrospective study, milk yield was negatively affected when cows were exposed to extreme THI conditions (> 77.78; Moore et al., 2024), which occurred only on one of the sampling days of this experiment. Therefore, mild to moderate heat stress conditions observed during the majority of the experimental period possibly were not sufficient to cause such metabolic alterations.

As expected, RT and RR exhibited a moderately positive correlation with each other, as well as with barn temperature and THI, as these physiological responses are known to be influenced by climatic variations (as reviewed by Becker et al., 2020). High relative humidity can exacerbate heat stress by impeding the rate of heat loss through evaporation (Silva et al., 2007). Contrary to expectations, moderate to very strong negative correlations were observed between relative humidity and RR, RT, THI, and barn temperature. Since the barn structure on the farm in this study was equipped with fan and sprinkler systems, the influence of this specific isolated variable may not have interfered as significantly as it would in an uncontrolled environment.

### Reproductive status

HS significantly impacts dairy cow reproduction, particularly under conditions of decreased nutrient intake and NEB. Thermic stress induces various hormonal changes, including those relevant to ruminant reproduction (Habeeb et al., 2018). Cortisol secretion increases under HS conditions, while levels of oestradiol, gonadotrophins-releasing hormone (GnRH), and luteinizing hormone (LH) decrease, thereby inhibiting estrus behavior (Sammad et al., 2020). Additionally, severe HS conditions can compromise the ovarian follicular environment and oocyte quality. NEB conditions may lead to hypocalcaemia and other metabolic issues directly related to uterine diseases or embryonic and fetal malformations (Paes et al., 2016).

This study found no significant differences in the reproductive status of cows with the use of mineral supplements. Notably, technological interventions such as sprinklers and fans, along with limited exposure to severe heat load, ensured balanced reproductive status between groups, alongside providing diets meeting nutrient requirements for the respective categories.

The thermoregulatory supplement comprised calcium, sodium, chlorine, and potassium, all of which are crucial nutrients for dairy cow reproduction (Min et al., 2019). Calcium plays a vital role in maintaining muscle contractibility and tone in the uterus, as well as promoting uterine involution. A deficiency in calcium can lead to reduced muscle contraction, resulting in declines in nutrient intake, rumen motility, and metabolic issues such as acidosis and ketosis (Ahuja and Pamar, 2017). Sodium and potassium are essential for maintaining osmotic balance, particularly important in combating HS (Desrousseaux, 2021). In cows exposed to more challenging environment and less optimal production settings, the mineral supplement may have more significant effects than those observed in this study.

### Limitations and practical implications

This experiment primarily focused on short-term effects and may benefit from longer-term observations to fully comprehend the sustainability of these improvements. Additionally, the sample size in this study was relatively small, and further research involving a larger population of dairy cows could offer more comprehensive insights. Access to and utilization of vaginal thermometers that provide a greater volume of body temperature data could enhance findings regarding mineral supplementation for preventing heat stress. However, due to financial constraints, evaluating metabolic parameters affected by heat stress was not feasible.

From a practical standpoint, the findings suggest that the use of a thermoregulatory mineral supplement, seeking to mitigate adverse effects of heat stress must be carefully evaluate according to each farm's conditions. Evaluating the integrated use of other available tools to prevent HS, such as housing, nutrition, and technology, may be more significant in maintaining overall wellbeing and productivity of their Holstein dairy cows.

Finally, the insights gleaned from this study on mitigating heat stress in Holstein dairy cows present a valuable opportunity to enhance agricultural education and knowledge dissemination. By integrating these findings into teaching programs and leveraging innovative tools, such as online resources and interactive workshops, we can empower a wide audience, including agricultural students, veterinarians, and dairy farmers, with practical strategies for managing heat stress effectively (Muca et al., 2023a,b). Collaboration with extension services and industry organizations further extends the reach of this knowledge, ensuring that it reaches farmers at the grassroots level.

## Conclusions

The use of thermoregulatory mineral supplement (containing calcium, sodium, chlorine, and potassium) did not significantly influence the physiological responses to HS or the reproductive status of Holstein cows. Although RR and RT showed a positive correlation with barn temperature and THI, regardless of experimental group, the climate conditions were not severe or prolonged enough to yield other significant results in this study.

## Declaration of generative AI in scientific writing

The authors did not use any artificial intelligence assisted technologies in the writing process.

## Ethical statement

The experiment did not include any invasive procedure in vivo; therefore, the research project was authorized as an observational study by the Ethical committee of the Centro de Ensino Superior Riograndense - CESURG, with protocol number 02.2023, date of approval October 20, 2023.

## Declaration of competing interest

The authors declare that they have no known competing financial interests or personal relationships that could have appeared to influence the work reported in this paper.

## Data Availability

Data reported in this study are available upon request to the authors.
